# The meiotic achilles’ heel: vulnerability and resilience under environmental stress

**DOI:** 10.1007/s00709-026-02201-1

**Published:** 2026-04-29

**Authors:** Attila Fábián

**Affiliations:** HUN-REN Centre for Agricultural Research, Agricultural Institute, Brunszvik 2, Martonvásár, 2462 Hungary

**Keywords:** Meiosis, Heat, Cold, Drought, Heavy metals, Oxidative stress

## Abstract

Plant meiosis constitutes a critical vulnerability under unfavorable conditions in plant reproduction. In the 21st century, with the accelerating global climate change, environmental stresses increasingly compromise fertility and genetic stability. This review examines the role of abiotic stress factors - including heat, cold, drought, salinity, heavy metals, and oxidative stress - in disruption of plant meiosis, the achilles’ heel of sexual processes. Heat stress appears particularly damaging, by compromising several molecular functions, such as DNA double-strand break formation, chromosome axis formation and spindle assembly. Cold stress affects meiotic recombination patterns as well as induces cytoskeletal disruption leading to unreduced gamete formation. While drought primarily impacts post-meiotic pollen development rather than meiosis itself, heavy metals cause widespread chromosomal abnormalities, generating reactive oxygen species and chromatin architectural alterations. In these stress responses, oxidative damage appears as a convergent pathway, yet paradoxically, controlled ROS production remains essential for normal meiotic progression. Critically, meiotic vulnerability is not merely an evolutionary oversight but potentially an adaptive feature. Stress-induced increasing recombination rate elevates genetic diversity, while weak checkpoints permit aneuploidy and unreduced gamete formation, thus facilitate polyploidization, a primary driver of plant speciation.

## Introduction

### Meiosis as a critical process in plant reproduction

Meiosis is an elemental cellular process, known since the 19th century (Hamoir 1992), and it is essential for the sexual reproduction of all eukaryotes, including flowering plants. Obviously, it is considered as the source of genetic diversity and evolutionary adaptation (Chen et al. 2025). The development of haploid gametes from diploid sporophyte cells starts with this specialized cell division, which reduces the chromosome number by half. The gametes then participate in fertilization to recreate the diploid state of offspring (Wang et al. 2021). However, critical importance of meiosis extends beyond simple chromosome reduction. It also includes complex mechanisms in order to ensure genetic recombination, proper chromosome segregation, and the maintenance of genome stability in subsequent generations (Wang and Copenhaver 2018).

As the first step of plant meiosis, plant chromosome axis forms during early leptotene as a proteinaceous structure built of meiosis-specific cohesin complexes, axis core proteins (ASY3 and ASY4), and HORMA domain proteins (ASY1 and ASY2) (Chambon et al. 2018; Ito and Shinohara 2023). This axis organization contains linear arrays of chromatin loops secured at their bases, which provides the essential scaffold for homologous chromosome recognition, pairing, and recombination (Ito and Shinohara 2023). Next, homologous chromosomes start a pairing process that ensures accurate genetic exchange. Complex molecular mechanisms help the recognition and alignment of homologous chromosomes (Lambing and Heckmann 2018). The recombination process starts with the formation of programmed DNA double-strand breaks (DSBs). This is a unique feature that distinguishes meiosis from mitotic division (Rafiei and Ronceret 2024). This controlled DNA damage provides a foundation for later events, which aim to create genetically diverse gametes. The synaptonemal complex (SC) forms between paired homologous axes during the zygotene stage, using the already present axes as lateral parts (Morgan et al. 2023). The addition of central components, including transverse filaments (ZYP1) and central element proteins (SCEP1, SCEP2, SCEP3) (Hesse et al. 2019; Vrielynck et al. 2023; Feng et al. 2025) completes the process. The SC ensures that chromosomes remain properly aligned during the extended prophase I period, allowing sufficient time for recombination machinery to process DSBs into mature crossovers (COs) (Gordon and Rog 2023). CO formation is considered as the completion of meiotic recombination because it creates physical links between homologous chromosomes. These links are necessary for proper segregation during the first meiotic division (Wang and Copenhaver 2018; Gutiérrez Pinzón et al. 2021). When crossing over is completed, two successive chromosome segregation events are needed to ultimately produce four haploid gametes from each diploid meiocyte (Cui et al. 2024). During the first division, homologous chromosomes separate, while the second division completes chromosome number reduction by sister chromatid separation. Meiosis helps sexually reproducing species to keep the constant number of chromosomes between generations. Recent research has revealed complex regulatory networks that control meiotic progression, including epigenetic modifications and post-translational protein modifications (Kuo et al. 2021). These controlling mechanisms ensure the temporal coordination of meiotic events as well as proper responses to environmental and developmental signals.

### Tissue-level organization and regulation of male and female meiosis

Majority of the available meiotic analyses focus on the easily accessible male meiosis, while the deeply enclosed female counterpart remains much less studied. As there are fundamental differences between the organization and development of the male and female germ units, these are discussed separately.

#### The male meiocyte-tapetum functional unit

In angiosperm anthers, male meiocytes (also known as pollen mother cells PMCs) and the surrounding tapetum layer work together as a functional unit. Here, structural connections between cells and the intensive intercellular signaling jointly regulate meiotic progression and gamete development (Lei and Liu 2020). PMCs are organized into so-called sporogenous archesporial columns (SACs) (Mursalimov et al. 2023). This structure works as a dynamic cellular continuum or functional coenocyte, as extensive cytoplasmic channels and plasmodesmata link the physiology of meiocytes and neighboring tapetal cells during prophase I (Heslop-Harrison 1966; Wang et al. 2006). Tapetal cells deliver several essential factors, such as metabolites, lipids, enzymes and redox buffering to developing PMCs (Lei and Liu 2020). Not surprisingly, disrupted tapetum development or flawed gene expression routinely leads to meiotic defects and ultimately, male sterility (Tidy et al. 2022). For instance, perturbation of the tapetum-specific transcription factor ABORTED MICROSPORES alters expression of meiotic genes in PMCs and disturbs radial microtubule arrays and callose deposition (Tidy et al. 2022). This underlines direct tapetum-meiocyte cross-regulation. In rice, premeiotic and meiotic callose deposition at PMC walls, mediated by GLUCAN SYNTHASE-LIKE5, is essential to time meiosis onset and maintain normal chromosome pairing (Somashekar et al. 2023).

#### Cytomixis and cytomictic channels

Cytomixis, the intercellular migration of nuclei or chromatin between neighboring male meiocytes, proceeds through enlarged cytomictic channels that differ from ordinary plasmodesmata by their diameter and frequency as well (Sidorchuk et al. 2007; Mursalimov et al. 2010). Serial block-face SEM of tobacco anthers reveals that 90–100% of normal meiocytes form over one hundred cytomictic channels to neighbors at leptotene-zygotene, and many nuclei extend protuberances through these channels without obvious damage (Mursalimov et al. 2021). Ultrastructural studies confirm that these channels result from fusion and dilation of pre-existing plasmodesmata or de novo wall perforation, showing irregular callose depositions (Mursalimov et al. 2019). Migrated chromatin retains SC structures and displays microtubule organizing activity to form minispindles (Mursalimov et al. 2019). Early ultrastructural work showed that cytomictic channels may occupy up to a quarter of the meiocyte interface, allowing organelles and ER elements to move between cells and causing the archesporial layer within a locule to behave as a giant coenocyte with shared cytoplasmic streaming (Heslop-Harrison 1966). In tobacco, cytomictic channels are distributed non-randomly over meiocyte surfaces and their number depends on meiotic stage, supporting nutrient equalization and synchronization but also propagating stress defects across the whole unit (Mursalimov et al. 2017).

#### Intercellular factors modulating male meiosis

The unique interconnected PMC-tapetum structure enables the high degree of synchrony of meiosis in PMCs and makes its regulation possible by external factors (Lei and Liu 2020). An example of such factors is the family of 24-nucleotide small interfering RNAs (24-nt siRNAs). After produced in tapetal cells by CLSY3-dependent RNA-directed DNA methylation (RdDM), 24-nt siRNAs are transported to meiocytes through cytoplasmic channels (Long et al. 2021). There, they trigger de novo DNA methylation, shape germline transcriptional programs and assist paternal epigenetic inheritance. Small RNA profiles of tapetal cells reflect those of meiocytes, and tapetal siRNAs can restore germline methylation patterns, even though RdDM biogenesis remains largely inactive within male meiocytes themselves (Lei and Liu 2020; Long et al. 2021). Beyond mobile RNAs, other factors, such as secreted proteins, lipid signals, oxygen and peptide hormones, as well as appropriate ROS gradients are also essential contributors provided by the tapetum for meiotic progression. Disruptions in tapetal redox balance or secretion often trigger chromosome mis-segregation and microspore abortion (Dukowic-Schulze and van Der Linde 2021). Callose dynamics at PMC walls and around cytomictic channels is indeed a major factor. Defective callose deposition both accelerates or desynchronizes meiotic entry and increases cytomixis, linking physical connectivity with checkpoint control (Sidorchuk et al. 2007; Somashekar et al. 2023).

#### Male versus female meiosis

In male meiosis, the PMC-tapetum continuum is a coenocytic and highly interconnected domain, where intercellular meiotic regulation is based on cytomictic channels and mobile siRNAs (Lei and Liu 2020; Long et al. 2021). By contrast, female meiosis occurs as a single megaspore mother cell (MMC) division, deeply embedded in the nucellus, and only a few reports are available on cytomixis, or broad cytomictic channels between the MMC and neighboring somatic cells (Petrella et al. 2021). Sporophytic control of female meiosis and the subsequent megagametogenesis is based largely on somatic small RNA (sRNA) pathways and plasmodesmatal communication between the MMC and nucellar as well as integumentary tissues. This can be well demonstrated by AGO9- and AGO5-dependent sRNA networks that restrict germline fate and promote functional megaspore development by intercellular signaling (Olmedo-Monfil et al. 2010; Tucker et al. 2012). Following meiosis, the female gametophyte transiently becomes a coenocytic embryo sac due to mitosis without cytokinesis. However, in contrast to the PMC-tapetum unit, this coenocytic phase is internal to the female gametophyte and does not involve direct cytomictic connection with surrounding somatic nurse cells (Petrella et al. 2021; Huang and Sun 2025). Overall, therefore, the male meiocyte-tapetum unit is a structurally coenocytic and cytomixis-dependent regulatory environment, whereas female meiosis is embedded in a more structurally isolated, largely sRNA-mediated signaling milieu, with important implications for how and what extent stresses perturb recombination and chromosome behavior in the two sexes (Lei and Liu 2020; Petrella et al. 2021).

### Vulnerability of meiosis to environmental stresses

**Fig. 1:**
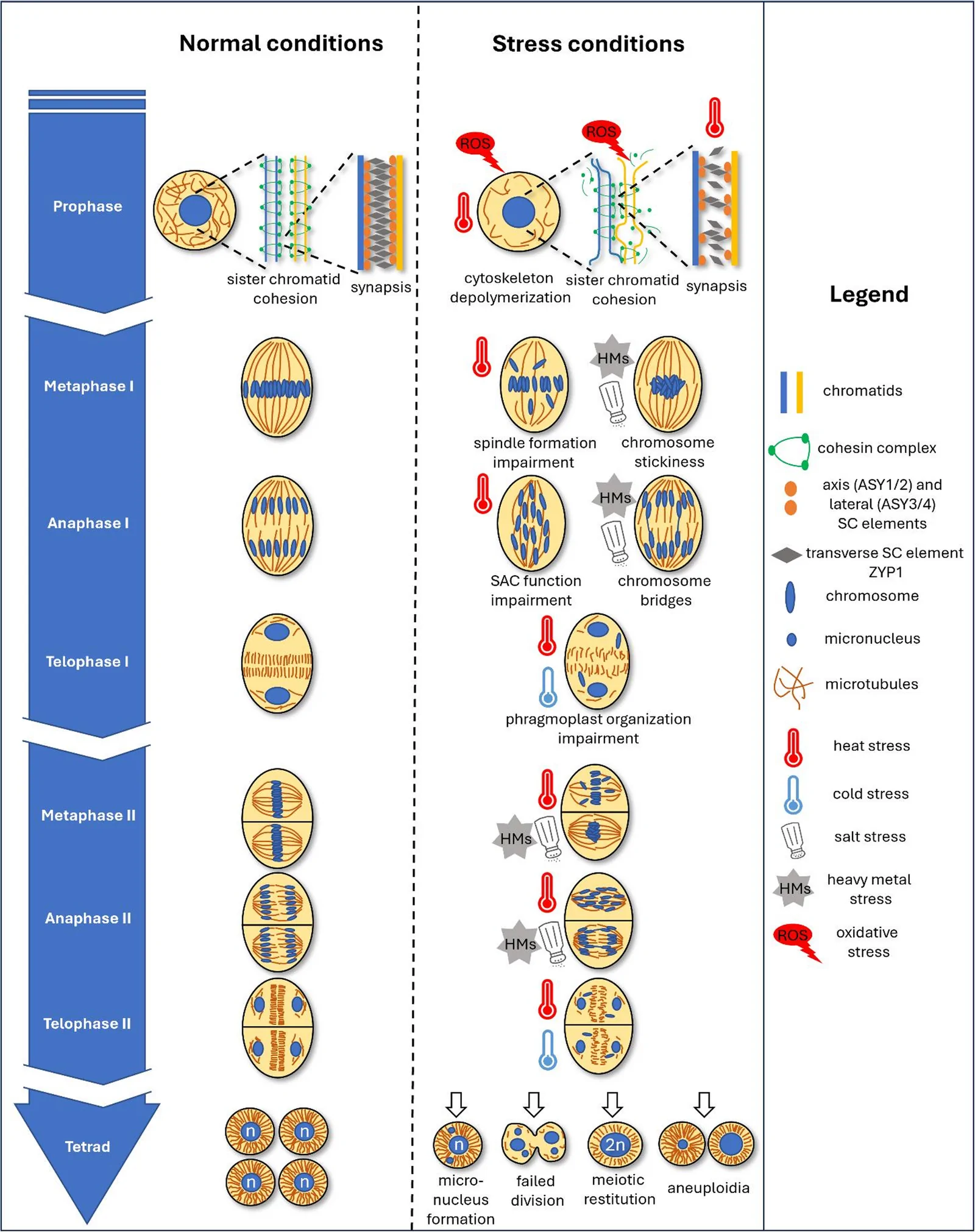
Unfavorable environmental factors cause negative effects during all stages of meiotic division. During prophase, sister chromatid cohesion and synapsis formation may be impaired. Later in metaphase and anaphase, the proper segregation of chromosomes is compromised due to structural defects in the spindle apparatus, malfunction of the spindle assembly checkpoint, or chromosome-associated abnormalities. In telophase, phragmoplast impairment may inhibit accurate cytokinesis. Various aberrations may occur in daughter cells, such as the formation of micronuclei, division errors, the occurrence of unreduced gametes, and the appearance of aneuploidy. Modified from Fábián et al. (2023), published in Frontiers in Plant Science under CC-BY 4.0 license

Considering the high complexity of meiosis, it is not surprising that this specialized cell division shows significant sensitivity to environmental perturbations. This vulnerability represents a bottleneck of sexual processes, where external stressors can seriously impact the behavior and structure of chromosomes, recombination patterns, and impair gamete formation. Environmental stresses incorporate a wide spectrum of factors including temperature extremes, drought, salinity, chemical pollutants, heavy metals, oxidative conditions, and anthropogenic compounds (De Storme and Geelen 2014; Bomblies et al. 2015). The meiotic process demonstrates particular susceptibility to these factors, especially when chromosomal pairing, synapsis, and CO formation occur during early prophase I. (De Storme and Geelen 2014). In addition, various structural problems are reported at the chromosomal level of DNA organization. The tightly regulated and cytoskeleton-intensive chromosome movements during meta- and anaphase, and phragmoplast construction at telophase are sensitive processes (Lei et al. 2020). Because of these multi-layered effects, a wide range of meiotic defects were discovered and described in the literature concerning the fields of plant stress and reproduction (Fig. 1). The environmental sensitivity of meiosis has extensive consequences for the whole sexual process. Stress-triggered alterations may lead to unreduced gametes, promote polyploidization, or even reproductive failure (De Storme and Geelen 2020; Crhak Khaitova et al. 2024). An important question arises: why evolutional processes left this critical vulnerability untouched? Is there a benefit underlying this seemingly critical weakness? Understanding the intricate system of regulatory and executive components standing behind the meiotic vulnerability, as well as the consequences of meiotic failure provides insights into plant fertility, adaptation mechanisms, and may also provide potential targets for crop improvement strategies (Lambing and Heckmann 2018; Kuo et al. 2021). This comprehensive review examines the effects of environmental stress on the intricate process of plant meiosis, focusing on stress-driven damage and molecular mechanisms.

## Effects of various abiotic stresses on meiosis

### Heat stress

Plant meiosis shows critical sensitivity to elevated temperature (for a summary, see Table 1), with different species showing distinct thermal thresholds for reproductive success. For instance, in *Arabidopsis thaliana* male meiosis, while optimal meiotic function occurs at approximately 16–21 °C, progressive dysfunction was observed when temperatures rose above 26 °C (De Storme and Geelen 2020; Crhak Khaitova et al. 2024). Complete fertility loss may occur around 30 °C in PMCs, and extreme temperatures of 36–38 °C trigger the catastrophic failure of meiosis (Lei et al. 2020). Thresholds for heat sensitivity show a remarkable species-specific variance, which points to the existence of distinct adaptation mechanisms instead of a single universal molecular mechanism. Although meiotic apparatus of *Arabidopsis* suffers severe damage at even 30 °C, certain plants-such as tropical cereals-maintain the functionality of meiosis at considerably higher temperature. This inter-species variability raises questions.

**Table 1:** Heat stress-induced damage to key meiotic- and spindle assembly checkpoint genes

Gene/Protein	Species	Locus ID	UNIPROT ID	Normal function	Heat stress effect	Temperature range	Mechanism of damage	Reference
AtSPO11	*Arabidopsis thaliana*	AT3G13170	Q9M4A2	Initiates DNA double-strand breaks (DSBs)	Reduced DSB formation	36–38 °C	Reduced protein activity leads to fewer DSBs	Ning et al. 2021
OsSPO11-1	*Oryza sativa ssp. japonica*	Os03g0752200	Q7Y021					Yu et al. 2010
AtCENH3	*Arabidopsis thaliana*	AT1G01370	A0A178WB57	Centromeric histone, kinetochore formation	Reduced protein levels at centromeres	26–30 °C	Decreased centromeric histone leads to weakened kinetochores	Crhak Khaitova et al. 2024
OsCENH3	*Oryza sativa ssp. japonica*	Os05g0489800	Q6T367					Liang et al. 2025
AtKNL2	*Arabidopsis thaliana*	AT5G025202	A0A1P8BHH1	CENH3 assembly factor	Increased heat susceptibility in mutants	30 °C	Decreased CENH3 stability	Ahmadli et al. 2023
γKNL2	*Oryza sativa ssp. japonica*	Os04g0347900	A0A8J8YDS3					Zuo et al. 2022
δKNL2	*Oryza sativa ssp. japonica*	Os01g0530300	A0A0P0V3K3					Zuo et al. 2022
AtBMF1	*Arabidopsis thaliana*	AT2G20635	A0A654F5C8	Kinetochore protein, chromosome attachment	Reduced protein levels at kinetochores	26–30 °C	Impaired kinetochore-microtubule attachment	Crhak Khaitova et al. 2024
AtBMF3	*Arabidopsis thaliana*	AT5G05510	A0A178UDA3	Spindle assembly checkpoint protein	Prolonged SAC activation	30 °C	Extended checkpoint duration delays meiosis	Crhak Khaitova et al. 2024
AtATM	*Arabidopsis thaliana*	AT3G48190	A0A1I9LQ79	DNA damage checkpoint kinase	Upregulated expression, specialized pachytene checkpoint	34 °C	Prolonged pachytene phase due to recombination monitoring	De Jaeger-Braet et al. 2021
OsATM	*Oryza sativa ssp. japonica*	Os01g0106700	n/a					Zhang et al. 2020
AtRAD51	*Arabidopsis thaliana*	AT5G20850	A0A178UQK2	DNA recombination and repair		36–38 °C	Impaired homologous recombination	Ning et al. 2021
OsRAD51A1, OsRAD51A2, OsRAD51C	*Oryza sativa ssp. japonica*	BGIOSGA035611 BGIOSGA037430 BGIOSGA013181	B8BLJ2 A2ZKR2 B8AN23					Tang et al. 2014; Liu et al. 2022
AtDMC1	*Arabidopsis thaliana*	AT3G22880	Q39009	Meiosis-specific recombinase	Reduced foci formation	36–38 °C	Impaired meiotic recombination	Ning et al. 2021;
TaDMC1	*Triticum aestivum*	TraesCS5A02G133000 TraesCS5B02G131900 TraesCS5D02G141200	A0A3B6KD55 A0A9R1HFM3 A0A9R1KZ01					Draeger et al. 2023
OsDMC1	*Oryza sativa ssp. japonica*	Os12t0143800-01	Q7GBF8					Ding et al. 2001
AtASY1	*Arabidopsis thaliana*	AT1G67370	F4HRV8	Axis associated protein	Premature destabilization	36–38 °C	Disrupted chromosome axis formation	Ning et al. 2021
OsPAIR2	*Oryza sativa ssp. japonica*	Os09g0506800	Q76CY8					Nonomura et al. 2004
AtASY3	*Arabidopsis thaliana*	AT2G46980	A0A7G2EHY4	Chromosome axis protein	Downregulated expression	36–38 °C	Compromised chromosome axis stability	Ning et al. 2021
OsPAIR3	*Oryza sativa ssp. japonica*	Os10g0405500	B9G5N1					Yuan et al. 2009
AtASY4	*Arabidopsis thaliana*	AT2G33793	A0A654EYK1	Chromosome axis protein	Destabilized chromosome axis, reduced expression	32–37 °C	Disrupted axis-SC connection	Fu et al. 2021
OsAM1	*Oryza sativa ssp. japonica*	Os03g0650400	Q53KW9					Che et al. 2011
AtZYP1	*Arabidopsis thaliana*	AT1G22260	A0A5S9VKH7	Central element of synaptonemal complex	Disrupted formation, aggregation	36–38 °C	Failed synapsis of homologous chromosomes	Ning et al. 2021
OsZEP1	*Oryza sativa ssp. japonica*	Os04g0452500	Q7FAD5					Wang et al. 2010
AtHSP101	*Arabidopsis thaliana*	AT1G74310	A0A5S9WUB5	Heat shock protein, protein folding	Critical for RAD51 loading under heat stress	32–35 °C	Without HSP101, RAD51 fails to load properly	Li et al. 2022
ZmHSP101	*Zea mays*	Zm00001eb293780	A0A804Q2Q4					
OsHSP101	*Oryza sativa ssp. japonica*	Os05g0519700	Q6F2Y7					Li et al. 2025 , b
Spindle assembly related genes (Aurora kinases, TPX2, formins, MAP65-3)	*Triticum aestivum*			Microtubule and actin nucleation, spindle assembly	Downregulated in heat-sensitive genotypes	35 °C	Impaired spindle organization	Fábián et al. 2023
Spindle assembly checkpoint (SAC) genes (MAD1, BUBR1, CDC20)	*Triticum aestivum*			Spindle assembly checkpoint	Downregulated in heat-sensitive genotypes	35 °C	Compromised SAC function leads to premature anaphase and aneuploidia	Fábián et al. 2023
TaPOK2	*Triticum aestivum*	TraesCS5A02G067300 TraesCS5B02G074200 TraesCS5D02G078500	A0A3B6KD66 A0A3B6LGY1 A0A3B6MLU7	Phragmoplast orienting kinesin	Affected by heat stress	35 °C	Impaired phragmoplast organization	Fábián et al. 2023
OsPOK	*Oryza sativa ssp. indica*	BGIOSGA035912	B8BMS7					Nishida et al. 2018
TAM/CYCA1;2	*Arabidopsis thaliana*, *Populus canescens*	AT1G77390 n/a	A0A178WDV8 n/a	A-type cyclin, meiosis I to II transition	Accumulates in stress granules, reduced protein levels	32 °C	Failed meiosis I-II transition leads to unreduced gametes	Zhou et al. 2022; De Jaeger-Braet et al. 2025
OsCYCA1;2	*Oryza sativa ssp. japonica*	Os01g0233500 Os12g0298950	Q7F830 A0A0P0Y9L4					Hao et al. 2014
AtCDKG1	*Arabidopsis thaliana*	AT5G63370	A0A654GDN4	Cyclin-dependent kinase; regulates cell cycle progression	Temperature-specific effects on crossover formation; fewer crossovers at high temperature	23 °C (effects), 12 °C (normal function)	Temperature-dependent regulation of crossover frequency	Zheng et al. 2014
OsCDKG1	*Oryza sativa ssp. indica*	BGIOSGA008570	A2X6X1					Tank and Thaker 2011

Concerning the general applicability of conclusions made using model organisms (such as *Arabidopsis*) and highlights the necessity of comparative studies. Temporal aspects of heat exposure are similarly critical. Heat stress during specific meiotic stages triggers lasting consequences. The premeiotic interphase to leptotene transition demonstrates a particularly vulnerable time window. In wheat, exposure to 30–35 °C for as short as 20 h during this critical window ceased normal meiotic progression, consequently reduced pollen grain number (Draeger and Moore 2017).

Temperature has significant effects also on meiotic recombination patterns and CO formation in plants. Studies show that both high and low temperatures increase CO frequency in *Arabidopsis* male meiocytes, and recombination rates follow a U-shaped curve (Weitz et al. 2021). The optimal level for recombination is near 18° C, while deviation in either direction alters CO frequency (Lloyd et al. 2018). Moderately elevated temperatures (20–28 °C) enhance crossing over formation through temperature-sensitive modulation of class I recombination machinery and altered synaptonemal complex dynamics, increasing Type I CO frequency by 10–15% (Modliszewski 2018; Lloyd 2018; Morgan 2017). Heat stress induces sex-specific responses in meiotic recombination. Brief temperature stress applied to female parents during reproductive stages has been shown to increase meiotic recombination rates in the progeny of *Arabidopsis*, whereas no such effect was observed in male parents (Saini et al. 2017).

In barley, elevated temperatures mainly increase recombination rates and shift crossover positions in PMCs, in parallel with longer synaptonemal complexes and more proximal Class I crossovers. In contrast, female recombination patterns remain largely unaffected (Phillips et al. 2015). This male-specific flexibility offers potential for temperature-based modulation in breeding.

Early prophase I is a critical window for establishing the chromosomal scaffold required for all subsequent meiotic events. The heat-induced disruption of axial protein complexes directly hinders homologous chromosome pairing and as well as crossover formation. It has been proven that damage to axis components severely disrupts meiotic progression. For instance, asy1 (asynaptic1) mutants show nearly complete loss of CO formation and failure of chromosome synapsis (Cuacos et al. 2021). Similarly, mutations in ASY3 or ASY4 gene trigger defective axis formation, as well as reduced CO numbers, and altered CO distribution patterns that favor distal chromosome regions (Chambon et al. 2018; Chu et al. 2024). These defects ultimately lead to chromosome segregation faults, inducing aneuploidy, and sterility. This sheds light on the importance of proper axis-SC coordination for the successful accomplishment of meiosis (Seear et al. 2020; Kursel and Rog 2024). Heat stress may simultaneously damage the synaptonemal complex and inhibit the expression of individual SC-associated proteins. ASY1 destabilizes prematurely in male meiosis while subjected to heat, inducing fragmented or incomplete axis formation (Ning et al. 2021). Accordingly, ASY4 is reported to become unstable in PMCs at temperatures above 32 °C (Ning et al. 2021; Fu et al. 2021; Zhao et al. 2025). These disruptions compromise the structural integrity of SC, which is necessary for proper chromosome pairing and synapsis - at least in male meiosis (Fu et al. 2021).

The transition to mid-prophase I requires precise molecular regulation of synapsis progression. CDKG1 (CYCLIN-DEPENDENT KINASE G1) controls recombination and chromosome synapsis processes (Hughes 2020). When CDKG1 function is lost during male meiosis, the synaptonemal complex forms incorrectly, and class I CO frequency is lowered (Zheng et al. 2014). Moreover, bivalent formation is severely compromised, and meiotic failure occurs ultimately in a temperature-dependent way (Zheng et al. 2014), suggesting the important role of the protein in heat sensitivity. The kinase stabilizes recombination intermediates early in the class I CO pathway, and it works upstream of ZMM (Zip1-4, Msh4-5, and Mer3) proteins (or synapsis initiation complex, SIC) to help proper pairing of homologous chromosomes while also preventing the chromosomes in PMCs from clumping during metaphase I (Lynn et al. 2007; Nibau et al. 2020).

During pachytene, the polymerization of ZYP1 into continuous linear arrays along chromosome pairs represents a critical quality-control checkpoint, as heat-induced aggregation of this central element protein directly prevents homologous chromosome synapsis and promotes univalent formation. Loading of the ZYP1-containing central element of SC is also vulnerable to heat stress. At temperatures of 36–38 °C, ZYP1 in male meiocytes was shown to deposit in aggregates rather than forming linear structures normally required for chromosome synapsis (Ning et al. 2021). This blocks proper pairing of homologous chromosomes and contributes to the formation of univalents in PMCs during meiosis I (De Storme and Geelen 2020; Lei et al. 2020).

Exposure to high temperatures activates specialized checkpoint mechanisms that monitor meiotic progression. For instance, the expression of ATM (Ataxia-Telangiectasia Mutated), a kinase protein mediator of a specialized pachytene checkpoint, elevates during male meiosis upon high temperature (De Jaeger-Braet et al. 2021). ATM is typically involved in DNA damage responses. This checkpoint is responsible for the quality control of the recombination process. When recombination intermediates are detected, extended duration of male meiosis is observed in *Arabidopsis* (De Jaeger-Braet et al. 2021; Zhao et al. 2023). Precisely put, pachytene phase prolonged at 34 °C, indicating the operation of this ATM-dependent checkpoint, which monitors recombination intermediates instead of general DNA damage (De Jaeger-Braet et al. 2021).

Elevated ambient temperature has been reported to impact several recombination-related molecular processes during leptotene and diplotene. As a consequence of heat stress, DNA double-strand break (DSB) formation becomes severely compromised: at temperatures of 36–38 °C in *Arabidopsis* male meiocytes, CO formation is completely abolished (Ning et al. 2021). Formation of programmed DSBs, a prerequisite for crossovers, relies on a multiprotein complex with several members (SPO11-1 and SPO11-2 meiosis-specific topoisomerases, DFO, MTOPVIB, PHS1, PRD1, PRD2, and PRD3; Thangavel et al. 2023). Surprisingly, only DFO expression showed a significant decrease in male meiosis under heat stress, while SPO11-1 and PRD1, PRD2, and PRD3 maintained stable expression levels (Ning et al. 2021; Zhao et al. 2023). Although expression data for MTOPVIB and PHS1 remain unavailable, these observations suggest that heat-induced DSB reduction may result from multiple factors in PMCs, including heat-triggered protein misfolding, disrupted chromosome axis assembly, and impaired topoisomerase complex localization (Ning et al. 2021). Remarkably, the meiosis-specific topoisomerase SPO11, which is responsible for initiating recombination by creating programmed DSBs, did not show a significant decrease in expression (Ning et al. 2021).

Another important function for successful recombination, DNA repair is also a subject of heat-driven influence. The loading of two essential meiotic recombinases, RAD51 (RADIATION-SENSITIVE 51) and DMC1 (DISRUPTION OF MEIOTIC CONTROL1), was severely reduced onto chromosomes during heat stress in male meiosis (Zhao et al. 2025), which potentially and at least partially originates from the above discussed DSB (that is, recombination substrate) number decline. RAD51 is a RecA-like recombinase that catalyzes the homology search and DNA strand invasion during homologous recombination (Bishop 2012; Xu et al. 2021). Homology search and strand exchange reactions are facilitated by the formation of nucleoprotein filaments on single-stranded DNA by RAD51. The protein is essential for both mitotic DNA repair and meiotic recombination, acting as an accessory factor for the meiosis-specific recombinase DMC1 (Lan et al. 2020). The expression of RAD51 was reported to decrease considerably in *Arabidopsis* male meiocytes after heat stress (Ning et al. 2021). As RAD51 appears to be regulated by DNA damage-dependent expression control in both somatic and meiotic cells (Nishizawa-Yokoi et al. 2023), it is not surprising that its levels decline when the number of programmed DSBs is reduced due to elevated temperature.

Thermal stress also efficiently triggers cytomixis during the final stage of prophase I in both dicotyledonous and monocotyledonous PMCs, for example, budding tobacco at 50 °C and air-dried barley at 48 °C producing comparable migration frequencies during early meiotic stages (Sidorchuk et al. 2016).

As metaphase begins, the centromere becomes a critical heat-stress vulnerability site. Centromere region structure and function are heavily affected by heat stress, triggering segregation failures during meiotic divisions. Accumulation of the centromeric histone variant, CENH3, is reduced at centromeres under elevated temperatures in male meiosis (Crhak Khaitova et al. 2024). As CENH3 protein gives the basis of kinetochore assembly, this effect has significant negative impact on segregation events. The reduction begins at moderate temperatures (26 °C) and becomes severe at 30 °C (Crhak Khaitova et al. 2024). Also, *Arabidopsis* mutant of the Kinetochore Null2 (KNL2) assembly factor for CENH3 showed increased heat susceptibility (Ahmadli et al. 2023), further supporting the important role of CENH3 in heat sensitivity of meiosis. The kinetochore protein BMF1 (BUB1/MAD3 FAMILY 1), which depends on CENH3 for proper localization, becomes undetectable at 30 °C in *Arabidopsis* PMCs (Crhak Khaitova et al. 2024). The resulting loss of kinetochore integrity stretches spindle assembly checkpoint (SAC) duration, which was evidenced by extended BMF3 kinetochore protein association with metaphase I kinetochores. The role of SAC is to examine kinetochore-microtubule attachments and block anaphase progression until all chromosomes are properly aligned and attached to spindle filaments (Komaki and Schnittger 2016).

The prolonged SAC duration reflects difficulties in achieving proper chromosome biorientation amidst thermal stress (Crhak Khaitova et al. 2024). Regarding gene expression, critical spindle assembly checkpoint (SAC) genes showed significant downregulation under thermal stress in wheat male meiocytes (Fábián et al. 2023). Aurora kinases, being essential for spindle organization and chromosome condensation, exhibit reduced expression after being treated at 35 °C. What is more, expression of a microtubule nucleation factor, TPX2 (targeting protein for Xklp2) protein, was downregulated in wheat (Fábián et al. 2023). This protein is also critical for proper spindle assembly (Vos et al. 2008). The core SAC component proteins MAD1 (MITOTIC ARREST DEFICIENT1), BUBR1 (BUDDING BY RELEASING 1-RELATED) and particularly CDC20 (CELL DIVISION CYCLE 20) showed reduced expression in the same experiment. The downregulation of these key genes compromises chromosome segregation fidelity and contributes to aneuploidy in male gametes.

The completion of chromosome segregation during anaphase I and progression through telophase I depend critically on the integrity of the cytoskeleton. Our earlier research in wheat showed that heat stress damages the cytoskeleton during male meiosis, causing extensive microtubule and actin filament depolymerization (Fábián et al. 2023). In a heat-sensitive wheat genotype, total microtubule length shortened by 44–59% in different meiotic stages, while a heat-tolerant variety possessed more stable cytoskeleton (Fábián et al. 2023). The consequences were faulty spindle assembly, and defective kinetochore fiber formation leading to narrower, fragmented spindles and laggard chromosomes. At the same time, many cytoskeleton-related genes had lower expression (Mozart-1, formins, MAP65-3), which shows the importance of these genes in meiotic heat stress sensitivity (Fábián et al. 2023). Chromosome segregation errors frequently arise during heat stress, including asynchronous chromosome movement in anaphase I, lagging chromosomes, and the subsequent formation of micronuclei. Overview of heat stress effects leads us to conclude the existence of a central susceptibility element: the cytoskeleton. Standing in the center of meiotic machinery, this is not merely one damaged system among many others, rather a node point where multiple forms of damage originate from. For example, heat damage of spindle elements and phragmoplast leads to different, yet equally serious consequences, such as micronucleus formation and even aneuploidy. Also, CENH3 depletion represents not simply a kinetochore problem, but leads to weakened microtubule binding sites, amplifying the effect of spindle instability. An interesting and important aspect of the temperature-stress-induced disruption of cytoskeleton and centromere integrity discussed above is the frequent formation of polyploids. Sharp changes in climatic conditions have been shown to lead to the repeated emergence of new polyploid species, highlighting the evolutionary significance of meiotic temperature stress sensitivity (Novikova et al. 2018).

Cell cycle regulation, affecting the transition between meiotic divisions, is indeed an important target for heat stress during meiosis. The TAM/CYCA1;2 cyclin, essential for the meiosis I to meiosis II transition, accumulates in stress granules under heat stress in PMCs of *Arabidopsis* to maintain adequate protein levels (De Jaeger-Braet et al. 2025). In contrast, heat stress reduces CYCA1;2 homolog expression leading to meiosis II failure and 2n pollen in Populus (Zhou et al. 2022). TAM/CYCA1;2 regulates TDM1 (THREE-DIVISION MUTANT1), which interacts with the APC and SMG7 (SUPPRESSOR WITH MORPHOGENETIC EFFECTS ON GENITALIA7, which regulates RNA decay), both critical meiosis exit regulators, that secure protein synthesis and meiotic progression (Bulankova et al. 2010; Cifuentes et al. 2016). Incorrect control of these components by TAM/CYCA1;2 can lead to the formation of unreduced gametes, because of the failure of meiosis II, which may induce polyploidization events (Zhou et al. 2022).

Heat-shocked cells activate a hierarchical network of transcriptional regulators and molecular chaperones that collectively function. Heat stress response is regulated by heat shock transcription factors (HSFs). These proteins serve as master regulators of thermotolerance by controlling the expression of numerous heat-responsive genes (Liu et al. 2011). Plants possess three distinct classes of HSFs (Classes A, B, and C), having different structural features and functional roles in heat stress signaling (Scharf et al. 2012; Andrási et al. 2021). While Class A HSFs work as transcriptional activators and are considered as central regulators orchestrating plant responses to heat stress, members of Class B are transcriptional repressors or co-activators, depending on the specific context (Guo et al. 2016). Heat shock proteins (HSPs), called to arms by HSFs, serve as main effector molecules of the heat shock response. These molecular chaperones have important protective functions, such as prevention from protein misfolding, facilitation of proper protein folding, and elimination of protein aggregates formed during heat stress exposure (Mondal et al. 2023). HSPs are classified into multiple families based on their molecular masses. Each HSP family owns unique mechanism of action and subcellular localization that contributes to thermotolerance. For instance, members of the HSP100 family, such as HSP101, promote the ATP-dependent disaggregation and reactivation of already aggregated proteins (Tiwari et al. 2021). HSP101 also facilitates RAD51 loading onto chromosomes in male meiocytes under heat stress. That is, its absence leads to severe fertility defects at elevated temperatures (Li et al. 2022). HSP90 family proteins function as co-regulators of heat stress-linked signal transduction complexes and manage protein folding in an ATP-dependent manner (Sangster and Queitsch 2005). HSP70 and HSP40 family proteins provide stabilization for newly synthesized proteins through ATP-dependent binding and release cycles (Hasanuzzaman et al. 2013; Usman et al. 2017). Small heat shock proteins (sHSPs) form a cellular matrix from high molecular weight oligomeric complexes in order to stabilize unfolded proteins. Subsequent release and refolding of their substrates requires the cooperation of HSP100, HSP70, and HSP40 family proteins (Hasanuzzaman et al. 2013; Bourgine and Guihur 2021). These diverse chaperone systems act together for protein protection and cellular recovery following heat stress exposure.

Although heat stress is the best-known meiotic damaging factor, there is a critical knowledge gap in current literature: insufficient understanding of meiotic recovery and acclimation mechanisms. While heat stress memory and thermotolerance induction are well-documented in vegetative tissues, these processes remain virtually unexplored in meiotic cells. The single live-cell imaging study investigating this phenomenon in male meiocytes found that damage of microtubule system is reversible at 30 °C, whereas at 34 °C it is not, suggesting a sharp threshold (De Jaeger-Braet et al. 2021). However, further studies are needed to examine whether repeated heat stress exposures can acclimatize meiotic cells against later stress. This knowledge is particularly important in the current climate change process, when plants frequently experience recurrent heat waves.

### Cold stress

Several meiosis-linked genes are reported to be cold-sensitive (summarized on Table 2). The recombination gene DMC1 appears to be a central factor in temperature tolerance during plant meiosis (Draeger et al. 2020). In PMCs of hexaploid wheat, the D-genome copy (TaDMC1-D1) is crucial for chromosome synapsis and CO formation in cold (Draeger et al. 2020, 2023). Loss of DMC1-D1 reduces COs by more than 95% when plants grow at 13 °C for 6–7 days. The cold sensitivity of DMC1 seems linked to expression level and protein stability as well in low temperatures (Draeger et al. 2020).

**Table 2:** Effects of cold stress on key meiotic genes and structural components

Gene/Protein	Species	Locus ID	UNIPROT ID	Normal function	Cold stress effect	Temperature range	Mechanism of damage	Reference
AtDMC1	*Arabidopsis thaliana*	AT3G22880	Q39009	Meiosis-specific RecA homolog; homology search and strand ex-change during meiotic recombina-tion	Deletion causes > 95% reduction in crossovers at 13 °C	13 °C (severe effects), 30 °C (moderate effects)	Reduced expression and compromised function lead to asynapsis, univalent formation, and crossover failure	Draeger et al. 2020, 2023; Ning et al. 2021
ZmDMC1	*Zea mays*	Zm00001eb163340	B4FMC1					
TaDMC1-D1	*Triticum aestivum*	TraesCS5A02G133000 TraesCS5B02G131900 TraesCS5D02G141200	A0A3B6KD55 A0A9R1HFM3 A0A9R1KZ01					
OsDMC1	*Oryza sativa ssp. japonica*	Os12t0143800-01	Q7GBF8					Ding et al. 2001
AtHEI10	*Arabidopsis thaliana*	AT1G53490	F4HRI2	Pro-crossover protein: marks sites destined to become class I crossovers	Increased foci formation at low temperatures	8 °C (increased activity), 18 °C (optimal)	Temperature affects HEI10 dynamics and crossover interference patterns	Lloyd et al. 2018; Kim et al. 2022
OsHEI10	*Oryza sativa ssp. japonica*	Os02g0232100	I6PL68					Tan et al. 2024
AtMLH1	*Arabidopsis thaliana*	AT4G09140	Q9ZRV4	MutL homolog; marks class I crossover sites and is required for crossover maturation	Reduced co-localization with crossover sites at low temperatures	8 °C (reduced function)	Impaired crossover maturation pathway affects meiotic recombination	Lloyd et al. 2018
OsMLH1	*Oryza sativa ssp. japonica*	BGIOSGA005217	B8A9J4					Xin et al. 2021
Radial Microtubule Array (RMA) components (Tubulin)	*Arabidopsis thaliana*			Organize cytokinesis in simultaneous-type meiosis	Formation severely disrupted by cold stress; leads to meiotic restitution	4–5 °C (disruption)	Compromised microtubule organization prevents proper cell wall formation	De Storme et al. 2012
Cold Shock Proteins (CSPs)	Various plant species			RNA chaperones; destabilize unfavorable RNA secondary structures at low temperatures	Induced by cold shock; facilitate translation and RNA processing	10 °C and below (induction)	Loss of function impairs RNA stability and translation efficiency	Sasaki and Imai 2012

Temperature has significant effects on meiotic recombination patterns and CO formation in plants. As discussed above a U-shaped thermal response curve exists with minimum recombination at physiologically optimal temperatures (18–23 °C) and elevated CO rates at both thermal extremes, as proven in male meiocytes (Morgan et al. 2017; Lloyd et al. 2018). Moderately reduced temperatures below the species optimum (8 °C versus 18 °C in *Arabidopsis* PMCs) increase crossing over frequency similarly to supraoptimal temperatures. However, effect of cold occurs through enhanced synaptonemal complex elongation and elevated class I crossovers including MLH1-independent pathways, further exemplifying the U-shaped response curve. MLH1 and HEI10 are key markers of class I crossover maturation. MLH1 localizes to mature class I crossovers destined for designation, while HEI10 marks crossover precursors and intermediates, which allows distinction between MLH1-dependent and MLH1-independent recombination outcomes across thermal conditions (Morgan et al. 2017; Lloyd et al. 2018). At elevated temperature (28 °C), both HEI10 and MLH1 foci increased significantly, whereas at reduced temperature (8 °C), MLH1 foci remained unchanged in male meiosis but CO numbers shifted toward MLH1-independent HEI10-marked COs. This demonstrates temperature-dependent plasticity in class I crossover maturation mechanisms (Lloyd et al. 2018; Kim et al. 2022). Low temperatures increase SC length in PMCs, thus provide more spatial substrate for recombination intermediate accumulation, particularly MLH1-independent HEI10-marked foci (Lloyd et al. 2018). Temperature-dependent modulation of SC architecture thus explains why elevated temperatures increase MLH1-marked crossovers while reduced temperatures increase HEI10-marked but MLH1-negative crossovers-both occur within the same class I pathway but through structurally distinct mechanisms (Morgan et al. 2017; Lloyd et al. 2018). These different responses suggest that plants use complex temperature sensing systems to adjust meiotic recombination.

Temperature extremes modulate class I, HEI10 dependent crossovers in *Arabidopsis* male meiocytes, which sheds light on a plastic recombination landscape that may facilitate adaptation to changing environments (Ziolkowski et al. 2017; Lloyd et al. 2018). Rather than disrupting meiosis, temperature fluctuations-both cold and heat-enhance the expressivity and flexibility of class I crossover formation, thereby boosting genetic diversity under stress (Lloyd et al. 2018; Modliszewski et al. 2018). This response shows evolutionarily advantage: elevated CO rates in PMCs under cold conditions facilitate rapid adaptation in short-season environments where population survival depends on genetic variation (Modliszewski et al. 2018). Similarly, increased CO formation at elevated temperatures, besides mechanisms like ATM-mediated DSB repair, provides enhanced male genetic diversity (De Storme and Geelen 2020; Zhao et al. 2023). A critical evolutionary insight may be derived from studies of enhanced recombination mutants in *Arabidopsis thaliana*. Genetic manipulation of CO formation-regulating genes, such as HEI10, RECQ4, FANCM, and FIGL1, does not compromise fertility or chromosome segregation accuracy despite substantially elevated CO frequencies in male meiosis (Ziolkowski et al. 2017; Serra et al. 2018). These genotypes maintain normal meiotic fidelity and low aneuploidy rates. Importantly, segregation errors under temperature stress originates from insufficient CO formation or defective repair rather than high numbers of crossovers (Modliszewski et al. 2018). This indicates that stress-induced enhanced crossovers boost adaptive potential without eroding meiotic accuracy. Concerning crossover interference, temperature perturbations do not compromise CO spacing regulation via the Type I pathway in PMCs (Modliszewski et al. 2018). Thus, stress-induced CO increases remain constrained by interference, which enables adaptive genetic variation, in parallel preserves segregation accuracy under heat and cold conditions.

Precise signaling and gene expression regulation are essential during meiosis to maintain chromosomal integrity and reproductive success under stress conditions. The involvement of gibberellin (GA) signaling in meiotic cold stress responses presents unresolved contradictions, contrasting with well-characterized vegetative pathways. The role of GA signaling in meiotic cold stress response remains contradictory and unresolved. While CBF1-CBF3 pathways clearly inhibit GA biosynthesis and stabilize DELLA regulator proteins in vegetative cold responses (Zhou et al. 2017), this connection has not been established in meiotic cells. Experiments with gai mutants (constitutive DELLA) demonstrated that cold-induced meiotic restitution is independent of DELLA levels in *Arabidopsis* PMCs (Liu et al. 2018). This fact may suggest either that meiotic cells employ a completely distinct temperature-sensing and response pathway, or that GA-DELLA signaling primarily drives vegetative cold responses, while meiotic cells rely on alternative signals. A critical gap exists in our understanding of which receptors and signaling cascades mediate temperature sensing in meiotic cells. Plasma membrane fluidity changes, calcium influx, or mechanosensitive channels are all candidates, yet these have barely been examined in meiotic contexts (Chinnusamy et al. 2004).

When plants face cold during meiosis, ICE1-CBF-COR gene cascade is one of the most important cold responsive regulative pathways (Hwarari et al. 2022). ICE1 (Inducer of CBF Expression 1) acts as a MYC-like bHLH transcription activator that binds to MYC sites in CBF (C-repeat Binding Factor) promoters (Chinnusamy et al. 2003). Under cold exposure, ICE1 stimulates CBF/DREB1 (Dehydration-Responsive Element Binding protein 1) transcription factors, which then induce the translation of cold-regulated (COR) genes (Hwarari et al. 2022). This signaling route is particularly important for meiosis, since many of its targets control cytoskeleton organization, membrane stability, and protective mechanisms that secure male meiotic accuracy in cold stress (Liu et al. 2017). Also, the expressed cold shock proteins (CSPs) are vital for RNA stability and translation efficiency in low temperatures (Sasaki and Imai 2012). These proteins act as RNA chaperones that remove faulty secondary structures of RNAs formed under cold influence. Consequently, CSPs are key players of freezing tolerance, and may defend meiosis indirectly, supporting gene expression and protein synthesis during cold stress.

In terms of cold impact on structural integrity, the most intriguing effect is selective cytoskeletal sensitivity characteristic for male meiosis. In contrast to heat-driven generalized microtubule destabilization throughout all meiotic phases, cold specifically damages telophase II radial microtubule arrays (RMA), leaving earlier spindle structures intact. In *Arabidopsis*, short exposure to 4–5 °C selectively blocks the formation of radial microtubule arrays (RMAs) at telophase II. This disruption causes defective cytokinesis and results in unreduced male gametes (De Storme et al. 2012). The resulting cold- induced meiotic restitution occurs during telophase II through second division restitution (SDR) mechanisms, in which disturbances of proper cell wall formation results in binuclear and polynuclear microspores that eventually develop into diploid and polyploid pollen grains (De Storme et al. 2012; Liu et al. 2018). The process sheds light on an important mechanism for sexual polyploidization in plants at low temperature stress conditions.

The phase-specific selectivity of cold-induced cytoskeleton impairment raises critical questions: (i) Why are earlier meiotic spindle microtubules insensitive to cold that destroys RMA? (ii) Are RMA-building tubulin isoforms distinct from those in metaphase I spindles? (iii) Or do differences between RMA construction mechanisms and meiotic spindle assembly explain differential thermal sensitivity? The literature provides no answers to these questions, despite being fundamental for completely mapping cold-induced unreduced gamete formation mechanisms. The agricultural significance of SDR - as a natural polyploidization pathway - further underscores the practical value of such knowledge (Liu et al. 2018, 2019).

The various effects of high and low temperature stress can be harnessed by researchers and breeders to generate new cultivars. Thermosensitive genic male sterility (TGMS) is among the most significant technologies in hybrid crop breeding, particularly in rice production (Pan et al. 2014). TGMS lines become male sterile at elevated temperatures (above 25–27 °C), while produce fertile pollen under lower temperatures (below 21–23 °C). This enables efficient two-line hybrid breeding systems (Wu et al. 2022), consequently provides an important tool in the hands of breeders. In contrast to TGMS lines, pollen of low temperature-sensitive genic male sterile (LTGMS) lines exhibit male sterility at cold conditions (e.g., below 24 °C). This difference offers a substitution to conventional TGMS lines and holds potential for hybrid breeding in cooler climates (Xu et al. 2023). Besides their breeding value, TGMS and LTGMS systems provide a unique platform for understanding temperature sensing and gene expression control at the molecular level (Wu et al. 2022). Numerous TGMS lines are known, linked to different genes and mechanisms (Wang et al. 2025a). While most target tapetum function, some of these lines have at least an indirect impact on meiocytes and meiotic progress (Table 3).

**Table 3:** Thermosensitive genic male sterility lines having impact on meiocytes and meiotic progress

TGMS line	Species	Gene name	Gene or transcript ID	UniProt ID	Meiotic function affected	Temperature range	Mechanism	Reference
Tian1S	*Oryza sativa ssp. japonica*	*OsMS1* ^*wenmin1*^	Os09g0449000	Q67V61	Indirect negative effect on meiosis progress through regulation of meiotic phasiRNA biogenesis	23 °C	Protein destabilization impairs transcription factor complex necessary for normal tapetum PCD	Lei and Liu 2020; Wu et al. 2022
PA64S	*Oryza sativa ssp. japonica*	*p/tms12-1*	osa-smR5864m	n/a	No direct effect on meiotic genes, impacts through impairment of tapetal functions	> 23.5 °C	Small RNA transcript regulates various transcription factors (MYB, TCP, and bHLH)	Zhou et al. 2012
BS366	*Triticum aestivum*	Kinesin	TraesCS2A02G480500 TraesCS2B02G505400 TraesCS2D02G479900	A0A3B5Y5I2 A0A3B6CDN3 A0A3B6DKL0	Components of phragmoplast, guide cell plate formation during cytokinesis	10 °C	Altered cytoskeletal organization disrupt cell division	Tang et al. 2011
		Profilin	TraesCS1A02G343400 TraesCS1B02G356700	A0A3B5Y5I2 A0A3B5Z1Y0				
		Formin	TraesCS6B02G163200	A0A3B6PHH4				
		ADF	TraesCS5A02G478500	A0A3B6KT12				
n/a	*Oryza sativa ssp. japonica*	*AGO1d (Argonaute 1 d)*	Os06g0729300	Q5Z5B2	No direct effect on meiotic progression; severe tapetal defects	< 22 °C	AGO1d in anther wall loads miR2118/miR2275 for phasiRNA biogenesis, the resulting tapetum failure impairs meiocyte support	Shi et al. 2024

OsMS1(MALE STERILITY1)-mediated sterility in Tian1S TGMS line impacts meiocyte development via defective programmed cell death (PCD) of tapetal cells, as the tapetum provides essential nutrients and structural materials to developing microspores (Wu et al. 2022). The OsMS1^wenmin1^ allele causes pollen sterility by delaying tapetum breakdown (Wu et al. 2022). At high temperatures, the OsMS1^wenmin1^ protein becomes unstable in the nucleus, weakening its binding to the TDR transcription factor, which controls genes essential for tapetum function and meiocyte support (Wu et al. 2022). This temperature-sensitive instability forms a thermosensitive regulatory system that disrupts activation of genes required for meiocyte development under heat stress (Wu et al. 2022). While there is no direct evidence for that OsMS1/TDR directly regulates meiotic recombination genes, the concept of tapetum dependent, non-cell autonomous control of meiosis, is well supported, in which tapetal transcription factors (UDT1, TIP2, TDR, EAT1) regulate meiotic phasiRNA (phased secondary siRNA) biogenesis and thereby support proper meiotic progression in PMCs (Lei and Liu 2020).

The *p/tms12-1* locus encodes a noncoding RNA precursor that yields the 21 nt small RNA osa smR5864w; a C→G single nucleotide polymorphism within this small RNA confers photoperiod sensitive genic male sterility in NK58S (PGMS line) and thermo photo sensitive sterility in PA64S (TGMS line) (Zhou et al. 2012). The mutation does not alter the sequence of any protein coding gene; instead, the variant small RNA is thought to target downstream transcripts, though the precise regulatory network remains under investigation. Cytological studies show that the p/tms12 1 allele causes abnormal tapetal development and microspore defects when plants are exposed to sterility inducing photoperiod or temperature during the microspore mother cell to meiosis window (Zhou et al. 2012). While downstream miRNA target networks involving MYB, TCP, and bHLH transcription factors have been implicated in PA64S fertility regulation (Wu et al. 2019), direct experimental evidence linking these networks specifically to the *p/tms12-1* small RNA is still lacking, and the locus does not directly regulate core meiotic recombination genes such as SPO11, DMC1, PAIR family members, or ZEP1 (Zhou et al. 2012). Cold stress severely disrupts male reproductive development in LTGMS wheat line BS366 by inducing critical defects in meiotic cytokinesis during meiosis I (Tang et al. 2011). When exposed to 10 °C during the pollen mother cell (PMC) and meiotic stages, the phragmoplast (the plant-specific microtubule apparatus essential for cell division) becomes aberrantly formed, preventing proper cell plate deposition and resulting in incomplete cell walls between the two developing dyads. At the molecular level, genes encoding the dynamic structural proteins essential for phragmoplast assembly and function (including profilin, formin isoforms, the kinesin KIFC2/KIFC3, and an actin-depolymerizing factor) are dramatically repressed (Tang et al. 2011). This cytoskeletal disorganization leads to failed cytokinesis during meiosis I and male sterility. This mechanism provides an example, how environmental temperature acts as a molecular switch controlling male fertility in LTGMS lines: brief cold exposure during the hypersensitive meiotic window specifically represses the dynamic cytoskeletal genes required for phragmoplast stability and centrifugal expansion, thereby triggering a cascading meiotic cytokinesis failure that leads to irreversible male sterility. AGO1d (ARGONAUTE1d) is expressed in various anther wall tissues, such as the epidermis, endothecium, middle layer, and tapetum during premeiotic and meiotic stages of rice (Oryza sativa) microsporogenesis (Shi et al. 2024). The protein associates with two microRNAs (miR2118 and miR2275) to trigger the biogenesis of 21 nt and 24 nt reproductive phasiRNAs (Shi et al. 2022b). Although ago1d knockout lines do not have visible effect on meiotic progression, the loss of AGO1d function and the resulting negative effect on the biogenesis of related phasiRNAs severely compromise somatic tapetal cell development, which is essential for supporting germ cell meiosis through provision of metabolites and nutrient reserves (Shi et al. 2022a).

### Drought stress

It is well supported that plants exhibit elevated sensitivity to water deficit conditions during the reproductive phase, leading to pollen sterility and reduced fertility (De Storme and Geelen 2014; Dietz et al. 2021). Nevertheless, when assessing effects of water scarcity, where stress conditions develop relatively slowly, proper timing of stress application as well as accurate assessment of its effects can be challenging for a relatively fast-occurring process such as the meiotic division. Consequently, although extensive studies have been carried out on the effects of drought on pollen development, relatively few gene expression- or proteomic analyses are available for the meiotic stage. During this narrow developmental window, water deficit induces severe structural problems in anthers. Data available concerning the effects of drought on gametogenesis derived mainly from microscopic analyses, revealing the appearance of defective anther locules, abnormal tapetal cells, and extensive pollen abortion in wheat and rice (Lalonde et al. 1997; Jin et al. 2013). Nevertheless, only limited data support the notion that drought directly impairs the process of meiotic division. Although reports in both rice (Namuco and O’Toole 1986) and pepper (Arnaoudova et al. 2021) described that water deficit can induce chromosomal abnormalities during male meiosis (e.g., perturbed spindle orientation and chromosome segregation, laggards, and micronuclei formation), other studies have not reported visible changes during meiotic division in drought treated PMCs of rice, wheat, or tomato (Saini and Aspinall 1981; Lalonde et al. 1997; Jin et al. 2013; Lamin-Samu et al. 2021). Jin and coworkers (Jin et al. 2013) conducted a comprehensive study of rice male meiocytes, in which they examined the expression of meiosis-specific genes following drought treatment. The genes analyzed in this experiment cover all major processes of meiotic division (chromosome condensation, cohesion and separation, chromosome pairing, synapsis, and recombination). Among the 77 genes investigated, only two, namely SPO11-2 and RAD51, showed a slight but significant increase in expression, while the vast majority of genes kept their stable expression following stress. Similarly, in drought stressed maize, no changes in meiosis-specific gene expression were observed, neither in the female ear (at floret formation stage) nor in the highly sensitive male tassel (undergoing meiosis), despite marked organ-specific transcriptomic differences (Zhuang et al. 2007).These findings lead us to conclude that the cause of drought-induced pollen abortion should be sought in later stages of development, after the completion of meiosis.

It is worth to note that studies of drought-stressed male development reporting abnormalities typically applied acute, serious water deficit (Namuco and OToole 1986; Arnaoudova et al. 2021), while others observing no direct effects used gradual water withholding, which is more realistic, standing closer to field conditions (Saini and Aspinall 1981; Jin et al. 2013). Such methodological discrepancies critically undermine comparative interpretation and highlight a persistent problem in stress physiology studies. This is the lack of stress intensity- and developmental window standardization. Moreover, a shift in preferred detection methods (microscopy-centrality of early studies versus the preference of new papers for molecular analysis) leads to missing information, as molecular and morpho-physiological alterations should be assessed in the same experiment. This methodological diversity raises a sobering possibility: meiotic drought plasticity may be more a viewpoint-derived artifact than biological reality.

When facing drought, plants readily accumulate various organic compounds to fight with osmotic disbalance and to protect macromolecules. These compounds, called osmolytes, include amino acids (proline), sugars (such as trehalose and sucrose), and quaternary ammonium compounds (glycine betaine) (Ghosh et al. 2021). Proline is one of the most important osmolytes, having multiple functions beyond osmotic adjustment, acting as molecular chaperone, antioxidant, and ROS scavenger (Man et al. 2011; Kavi Kishor et al. 2022; Khan et al. 2025). Studies confirmed that proline is required for male gametogenesis and accumulated in pollen grains at high concentration (Mattioli et al. 2012; Biancucci et al. 2015), which may partly explain the relative high drought resistance of gametogenesis. Biosynthesis of proline becomes highly active when drought and heat stress occur, contributing to the maintenance of cellular redox balance through the NADP+/NADPH ratio and defending the photosynthetic apparatus from photoinhibition and oxidative damage (Bandurska et al. 2017; Kavi Kishor et al. 2022). Moreover, as proline metabolism shows subcellular compartmentalization (synthesized in the cytoplasm but catabolized in the mitochondria), it is very likely that not only the accumulation, but the cycling of this compound may also be crucial (Verslues and Sharma 2010). Could proline catabolism in mitochondria serve as an alternative electron donor for ATP generation during the high energy-demanding meiosis in drought? Although role of proline as alternative respiration substrate is established (Launay et al. 2019), this hypothesis needs testing. However, it provides an enticing explanation for the apparent proline necessity for successful pollen development under stress conditions.

### Salt stress

Evaluation of salt stress effects on sexual processes of plants is a relatively less studied field compared to other environmental issues. Nevertheless, available research in *Arabidopsis* demonstrates that salinity-induced osmotic stress adversely affects reproductive development: it inhibits microsporogenesis and stamen filament elongation, enhances programmed cell death in the chalaza and integuments, and causes extensive ovule abortion (Sun et al. 2004). A study on Triticum turgidum plants exposed to 100–175 mM NaCl reported significant reduction in several reproduction-linked metrics. Decreased spikelet number per spike, as well as delayed spike emergence were observed, which may subsequently result in poor grain yields (Munns and Rawson 1999). Important to note, these effects occurred even when Na⁺ and Cl⁻ concentrations in shoot apex tissues were below levels that would directly limit metabolic reactions. Research using *Arabidopsis* plants demonstrated that exposure to NaCl -along with heat, cold, UV light and flooding- resulted in an increase in somatic homologous recombination frequency (HRF) (Boyko et al. 2010). This universal effect shows that HRF increasement is rather a general adaptive response for unfavorable conditions than a specific mechanism for salt exposure. However, salt-driven elevation of recombination frequency seems linked to DNA damage caused by salt-induced oxidative stress. Salt treatment leads to elevated production of reactive oxygen species (ROS), which cause DNA double-strand breaks (Dmitrieva et al. 2011). These stress-driven DNA lesions possibly interfere with normal, programmed DSBs, directly influencing meiotic stability. General chromosomal problems (such as chromosome stickiness, bridges, laggards and formation of micronuclei) were also reported in dividing cells of Allium bulbs after 0.15 M NaCl treatment (Çavuşoğlu 2023). However, it would be important to test the chromosome-damaging nature of high saline concentration also on meiotic cells.

### Heavy metal stress

Heavy metals (HMs), including lead (Pb), cadmium (Cd), mercury (Hg), zinc (Zn), arsenic (As), copper (Cu) and chromium (Cr) have very diverse and extensive effects on plants (Kiran et al. 2022). The main effect is the generation of reactive oxygen species (ROS) (Moura et al. 2012; Dutta et al. 2018). Through this, HMs trigger various damages in cellular macromolecules, such as proteins, lipids, nucleic acids (Nagajyoti et al. 2010). Obviously, meiotic processes show vulnerability to heavy metal triggered damage, as can be seen on Table 4, showing a wide spectrum of chromosomal abnormalities as effects of HMs.

**Table 4:** Effects of heavy metals on meiosis

Heavy metal	Effect/Concentration range	Mechanism of damage	Species	Reference
Mercury (Hg)	Decreased pollen fertility (72% at 80 ppm)	Chromosome stickiness, bridges, laggards	*Triticum aestivum*	Rai and Dayal 2016
Mercury (Hg)	Greater genotoxicity than Cd (32.82% abnormalities at 400 ppm)	Stickiness, fragmentation, bridges, micronuclei	*Glycine max*	Kumar and Rai 2007
Mercury (Hg)	Severe chromosome stickiness (4.95% at 400 ppm)	Chromosome clumping, spindle dysfunction	*Vicia faba*	George 2000
Mercury (Hg)	Decreased pollen fertility (79% at 100 ppm)	Chromosome stickiness, bridges, laggards, fragments	*Zea mays*	Kumar Rai and Kumar 2010
Lead (Pb)	Decreased pollen fertility (60.6% at 300 ppm)	Chromosome stickiness, bridges, laggards, disturbed polarity	*Lathyrus sativus*	Tripathi and Girjesh 2010
Lead (Pb)	Chromosome abnormalities (15.62% height reduction at 100 ppm)	Frequent cytomixis, micronuclei, desynapsis	*Capsicum annuum*	Aslam et al. 2017
Cadmium (Cd)	Decreased pollen fertility (64.6% at 300 ppm)	Precocious chromosome movements, laggards, non-orientation	*Lathyrus sativus*	Tripathi and Girjesh 2010
Cadmium (Cd)	Total meiotic abnormalities (19.24% at 300 ppm)	Chromosome stickiness, bridges, disturbed phases	*Vicia faba*	George 2000
Cadmium (Cd)	Decreased pollen fertility (77% at 100 ppm)	Chromosome stickiness, bridges, laggards, fragments	*Zea mays*	Kumar Rai and Kumar 2010
Cadmium + Chromium (Cd + Cr)	Reduced pollen fertility and meiotic abnormalities	Multiple meiotic abnormalities	*Hordeum vulgare*	Mittal and Srivastava 2014
Zinc (Zn)	Lower toxicity than Pb (19.24% abnormalities at 300 ppm)	Secondary associations, stickiness, laggards	*Lathyrus sativus*	Tripathi and Girjesh 2010
Lead+Zinc (Pb + Zn)	Synergistic effects on meiotic abnormalities	Chromosome stickiness, B-chromosomes, laggards, increased cytomixis	*Stachys inflata*	Hajmoradi and Taleb Beydokhti 2019

Vast majority of our understanding on HM-generated damage on meiosis is from male meiocytes. The most common defect induced by HMs is chromosome stickiness, the formation of compact chromatin masses that lose their individual identity (George 2000; Tripathi and Girjesh 2010; Aslam et al. 2017). This effect originates from the partial dissociation of nucleoproteins and alteration in chromosome organization patterns (Kumar and Rai 2007).

A similar phenomenon, chromatin agglutination, may occur when metal ions alter chromatin architecture. Chromatin agglutination manifests in the form of chromosome clumping as well as persistent chromosomal bridges across several meiotic stages (Mittal and Srivastava 2014). Another major effect is spindle apparatus dysfunction, where HMs disrupt spindle formation and activity, resulting in improper chromosome segregation, lagging chromosomes, and disturbed polarity (George 2000; Hajmoradi and Taleb Beydokhti 2019). Such abnormalities frequently lead to the formation of micronuclei (Kumar and Rai 2007; Aslam et al. 2017). HM pollution can also elevate cytomixis rates (Hajmoradi and Taleb Beydokhti 2019). Furthermore, HMs reduce the frequency of chiasma formation as well as damage accurate chromosome pairing and genetic recombination during meiosis (George 2000). While the literature predominantly focuses on HM effects in male meiocytes, a publication on metallophyte *Cardaminopsis arenosa* from Zn/Pb/Cd-polluted sites revealed female sensitivity. In this study, although male meiosis and pollen were largely unaffected, with low necrosis, female meiocytes showed 23.5–28% abnormalities (e.g., degeneration of megaspore mother cells, dyads, and tetrads) (Kwiatkowska and Izmaiłow 2014).

Plants possess various and effective defense and tolerance mechanisms to cope with heavy metal toxicity (Emamverdian et al. 2015). Metallothionein proteins and cysteine-rich oligopeptides, called phytochelatins serve as molecular chelators for heavy metal detoxification in plants. High level expression of metallothioneins was confirmed in the anthers and ovules of *Arabidopsis*, as well as in the tapetum of maize during meiosis (Fordham-Skelton et al. 1997; Charbonnel-Campaa et al. 2000). This indicates the important role of these proteins in meiotic HM resilience of both sexes. Cell wall sequestration, vacuolar compartmentalization, and antioxidant systems are additional protective strategies (Mansoor et al. 2023; Faizan et al. 2024; Sreelatha et al. 2025). The cell wall represents the first line of defense against HMs, acting as both a barrier to metal entry and a target for metal accumulation through ion exchange and chelation processes. Cell walls are modified after HM exposure to increase their capacity to bind metal ions while reducing permeability to protect the protoplast (Krzesłowska 2011). Mycorrhizal associations may further enhance plant HM tolerance by immobilizing these compounds in fungal cell walls and reducing metal uptake (Upadhyaya et al. 2010).

A poorly understood aspect of meiotic HM defense mechanisms is the spatial localization and temporal expression of the involved molecules. Metallothioneins have been detected at high levels in anthers and tapetum of several species during meiosis (García-Hernández et al. 1998; Charbonnel-Campaa et al. 2000; Yi et al. 2016), which may indicate the existence of meiosis-specific mechanisms against HM-induced damage. However, several critical questions should still be answered: are these metallothioneins expressed constitutively or induced by HMs? Is there a correlation between their expression and the level of meiotic HM resilience? More fundamentally, how does the temporal coordination of phytochelatin synthesis and metallothionein expression align with the progression through vulnerable meiotic stages?

### Effects of oxidative stress on plant meiosis

Oxidative stress (OS) is a major threat for meiotic processes in plants. OS occurs when production of reactive oxygen species (ROS) exceeds the detoxifying cellular antioxidant capacity (Sachdev et al. 2021; Hasanuzzaman and Fujita 2022). Plant meiosis is particularly vulnerable to oxidative damage due to its complex molecular machinery (Hörandl and Hadacek 2013; Perkins et al. 2016). Reactive oxygen species (ROS) ˗ including hydrogen peroxide (H₂O₂), superoxide anions (O₂^−^), hydroxyl radicals (OH^−^), and singlet oxygen (¹O₂) ˗ are continuously generated as byproducts of normal cellular metabolism. ROS concentrations may rise dramatically at environmental stress (Czarnocka and Karpiński 2018). These compounds inflict significant damage on plant cells by oxidizing macromolecular building blocks like proteins, lipids and nucleic acids (Juan et al. 2021). While the damaging potential of ROS was recognized early, it has since become obvious that these molecules fulfill indispensable roles in cellular stress sensing, signaling, and regulatory networks (Mittler 2017). When considering meiosis, a similar duality of ROS is present, being simultaneously both a blessing and a curse. While stress-induced oxidative damage is deleterious, many reactive oxygen species are recognized as crucial for normal reproduction at physiological concentrations (Kurusu and Kuchitsu 2017; Breygina and Klimenko 2020; Sankaranarayanan et al. 2020, 2025).

#### Generation of oxidative stress by abiotic stresses

Both high and low temperatures induce oxidative stress, however, the mechanisms laying in the background are different (Hasanuzzaman et al. 2013). *High temperature* generates OS in plants primarily by the disruption of cellular membranes and electron transport chains in chloroplasts and mitochondria. This damage leads to excessive ROS production (Suzuki and Katano 2018). Additional way for OS generation is the heat-induced calcium accumulation in mitochondria, which causes hyperpolarization of the inner mitochondrial membrane, promoting additional ROS generation (Rikhvanov et al. 2014). Also, heat-driven damage to photosystem II in chloroplasts, particularly the D1 protein and oxygen-evolving complex, results in incomplete electron transport and accumulation of singlet oxygen and hydrogen peroxide (Yamashita et al. 2008). On the contrary, when plants are exposed to cold stress, membrane rigidification and cold-induced imbalance between photosynthetic electron transport and the Calvin-Benson cycle are key factors in ROS generation (van Buer et al. 2019; Dong et al. 2025). Cold may also induce ROS formation via activation of a calcium channel, elevating cytosolic Ca levels, as it is reported in human cells (Sun et al. 2016).

*Cold stress* delivers its effects not only by the production of ROS, but also by impairing ROS detoxification: suboptimal temperatures lower the stability of proteins and protein complexes, leading to reduced activity of ROS scavenging enzymes (Yu et al. 2025).

*Drought stress* affects ROS homeostasis via multiple mechanisms (Cruz De Carvalho 2008). Stomatal closure instantly limits CO₂ fixation and reduces NADP⁺ regeneration in the Calvin cycle. This triggers over-reduction of the photosynthetic electron transport chain. Increased electron leakage to oxygen occurs via the Mehler reaction (Miller et al. 2010). Drought stress also reported to impair antioxidant enzyme activities, while simultaneously increasing ROS production. This may create a cycle of oxidative damage (Cruz De Carvalho 2008; Zhang et al. 2021).

*High salinity* disrupts ion homeostasis and causes osmotic stress. The resulting metabolic imbalances enhance ROS generation inside the cells (Kesawat et al. 2023). Certain NADPH oxidase genes like AtRbohD and AtRbohF (*Arabidopsis thaliana* Respiratory Burst Oxidase Homolog) show rapid expression increases after salt treatment (Ma et al. 2012). Furthermore, the enzyme glucose-6-phosphate dehydrogenase is significantly stimulated during salt stress. This provides NADPH substrate for NADPH oxidase activity and ROS generation (Nemoto and Sasakuma 2000). An additional effect of salt stress is the direct damage of chloroplast membranes and disruption of the photosynthetic electron transport chain. This also leads to increased ROS production.

*Heavy metals* also induce oxidative stress (Ercal et al. 2001; Nowicka 2022). Redox-active metals, e.g. Fe, Cu, and Cr directly participate in Fenton and Haber-Weiss reactions and generate hydroxyl radicals. Others as Pb, Cd, and Hg rather deplete cellular antioxidant pools, particularly the thiol-containing compounds like glutathione, and also inhibit antioxidant enzymes (Balali-Mood et al. 2021). Cr(IV) triggers ROS production at high concentrations, seriously impairing the DNA- and lipid-containing structures of cells (Balali-Mood et al. 2021). Although most environmental stresses harm plant cells at least partly via oxidative processes, only a relatively few reports are available on the specific impacts of ROS on sexual processes, and particularly on meiosis itself.

#### Effects of oxidative stress on meiotic processes

Oxidative stress strongly impacts plant meiosis at multiple stages. For instance, inefficient processing of damaged DNA sites in premeiotic S phase or early prophase I in plant reproductive tissues generates secondary strand breaks that may enter meiotic HR pathways. Abasic (AP) sites arise spontaneously from depurination or depyrimidination and as obligatory BER intermediates after glycosylase action on ROS modified bases (Fortini et al. 2003; Svilar et al. 2011). These are repaired mainly via base excision repair (BER) and single strand break repair (SSBR): AP endonucleases (APE1 like proteins) or AP lyases incise the backbone, followed by end processing, DNA synthesis and ligation (Svilar et al. 2011; Hegde et al. 2012). Because AP sites are chemically unstable, they can undergo β elimination or be cleaved by AP endonucleases to generate SSBs, especially in highly metabolically active or condensed chromatin (Fortini et al. 2003; Hegde et al. 2012). In plant reproductive tissues, inefficient processing of AP sites during pre-meiotic S phase or early prophase I increases the likelihood of secondary strand breaks. These breaks must be handled using DSB repair pathways, including meiotic homologous recombination (HR) (Manova and Gruszka 2015; Szurman-Zubrzycka et al. 2023). Thus, AP site homeostasis is an important upstream determinant of the DSB load experienced by meiotic recombination machinery during oxidative stress (Hegde et al. 2012; Szurman-Zubrzycka et al. 2023).

ROS also directly attack the deoxyribose-phosphate backbone during premeiotic stages. This leads to the generation of single strand breaks (SSBs) and sugar fragmentation products with blocked 3′ or 5′ termini (Hegde et al. 2012; Cadet et al. 2017). Many BER intermediates also manifest transiently as SSBs (Hegde et al. 2012). SSBR, which extensively overlaps with BER, repairs these lesions via end processing (e.g. AP endonucleases, phosphodiesterases), gap filling and ligation, scaffolded by factors such as PARP and XRCC1 like proteins in both animal and plant systems (Hegde et al. 2012; Spampinato 2017). When SSBs occur in close proximity on opposite strands or are encountered by replication or transcription complexes, they can be converted into one ended or two ended DSBs. These become substrates for HR or non-homologous end joining (NHEJ), respectively (Hegde et al. 2012; Cadet et al. 2017). In plants, defects in BER/SSBR components increase sensitivity to oxidative stress and elevate DSB levels, therefore increasing the demand on meiotic HR when ROS-derived damage occurs in meiotic cells (Manova and Gruszka 2015; Szurman-Zubrzycka et al. 2023). Consequently, the efficiency of SSBR strongly influences the level of ROS-induced damage that eventually appears to meiotic recombination machinery as DSBs that must be repaired using homologous chromosomes (Wright et al. 2018; Emmenecker et al. 2023).

When considering oxidative damage affecting meiosis, we must not overlook DNA lesions that may directly impact homologous recombination. ROS frequently oxidize nucleobases to form 8-oxo-7,8-dihydroguanine (8-oxoG), formamidopyrimidines and oxidized pyrimidines such as thymine glycol, which are generally non-bulky and weakly helix-distorting (David et al. 2007; Cadet and Wagner 2013). These lesions are predominantly repaired by BER, initiated by lesion-specific DNA glycosylases (OGG1/FPG-like for 8-oxoG/Fapy, NTH/NOG-like for oxidized pyrimidines), followed by AP endonucleases, gap filling synthesis and ligation (David et al. 2007; Manova and Gruszka 2015). Plant genomes encode functional orthologs of these BER components and BER is essential for maintaining genome integrity under chronic oxidative stress in meristems and reproductive tissues (Manova and Gruszka 2015; Spampinato 2017).

Clustered or bistranded lesions may be combinations of oxidized bases, AP sites and SSBs within one or two helical turns. These are characteristic hallmarks of ROS damage and are highly prone to conversion into DSBs (Cadet and Wagner 2013; Cadet et al. 2017). Once formed, DSBs are processed by resection and then repaired via homologous recombination (HR), classical non homologous end joining (c NHEJ), or alternative end joining/single strand annealing, with pathway choice determining mutational versus recombinational outcomes (Puchta 2005; Wright et al. 2018). In somatic plant cells, c NHEJ is often dominant, but is strongly favored during meiosis. HR employs RAD51 and DMC1 recombinases, together with numerous accessory factors, to repair both Spo11 induced and accidental ROS induced DSBs (Schwarzacher 2003; Emmenecker et al. 2023). In meiosis, only a minority of HR repair events are resolved as crossovers, with most yielding non-crossover gene conversions, so ROS-derived DSBs primarily increase the burden of high-fidelity repair rather than dramatically boosting crossover numbers (Schwarzacher 2003; Hörandl and Hadacek 2013). However, under severe oxidative stress, disrupted DSB formation and HR repair can overwhelm meiotic progression in plant meiocytes, leading to chromosome fragmentation, mis-segregation and reduced fertility, phenomena frequently observed in stressed male reproductive development (De Storme and Geelen 2014).

Certain ROS-dependent reactions generate helix-distorting lesions such as 5′,8-cyclo-2′-deoxypurines, lipid peroxidation-derived adducts and DNA-protein crosslinks, which are less efficiently handled by BER and instead are preferential substrates for nucleotide excision repair (NER) and, in some cases, HR (Svilar et al. 2011; Shafirovich and Geacintov 2017). Plant NER machinery, including XPC-, TFIIH-, XPA- and XPF/ERCC1-like factors, is conserved and can remove these bulky oxidative products by dual incisions flanking the lesion followed by gap filling synthesis and ligation (Manova and Gruszka 2015; Spampinato 2017). Certain ROS-dependent reactions generate helix-distorting lesions such as 5′,8-cyclo-2′-deoxypurines, lipid peroxidation-derived adducts and DNA-protein crosslinks, which are less efficiently handled by BER and instead are preferential substrates for nucleotide excision repair (NER) and, in some cases, HR (Svilar et al. 2011; Shafirovich and Geacintov 2017). Plant NER machinery, including XPC-, TFIIH-, XPA- and XPF/ERCC1-like factors, is conserved and can remove these bulky oxidative products by dual incisions flanking the lesion followed by gap filling synthesis and ligation (Manova and Gruszka 2015; Spampinato 2017). Stalled replication or transcription complexes at bulky lesions or crosslinks in pre-meiotic S phase are often processed into DSBs, which then enter HR pathways that are subsequently reused in canonical meiotic recombination (Wright et al. 2018; Emmenecker et al. 2023). Such lesions and their NER/HR-mediated processing may alter local chromatin structure and topological stress, thereby influencing Spo11 access and shifting the distribution or efficiency of meiotic DSBs and crossovers in plant genomes (Hörandl and Hadacek 2013; Manova and Gruszka 2015). Overall, bulky oxidative DNA damage constitutes a quantitatively smaller but mechanistically important class of ROS lesions linking NER and HR with meiotic recombination, especially under extreme stress conditions (Spampinato 2017; Shafirovich and Geacintov 2017).

Such lesions and their NER/HR-mediated processing may alter local chromatin structure and topological stress, thereby influencing Spo11 access and shifting the distribution or efficiency of meiotic DSBs and crossovers in plant genomes (Hörandl and Hadacek 2013; Manova and Gruszka 2015). Overall, bulky oxidative DNA damage constitutes a quantitatively smaller but mechanistically important class of ROS lesions linking NER and HR with meiotic recombination, especially under extreme stress conditions (Spampinato 2017; Shafirovich and Geacintov 2017).

In this light, the ROS generated damage described above can be viewed not only as threats to genome stability in modern plant meiocytes, but also as a factor that may have driven the evolution of meiotic homologous recombination as a dedicated high fidelity repair pathway. On a physiological level, diverse abiotic stresses manifest as oxidative stress, and repair of ROS induced DNA damage has been proposed as a major driving force in the evolution of meiosis (Hörandl and Speijer 2018). Building on this concept, Hörandl and Speijer argued that endogenous ROS production by the proto mitochondrion and the resulting oxidative DNA damage in early eukaryotes could have triggered the emergence of meiosis-mixis cycles as a specialized homologous recombination-based repair strategy (Hörandl and Speijer 2018). This evolutionary perspective suggests that the meiotic recombination machinery currently coping with Spo11 induced and ROS derived double strand breaks in plant meiocytes may itself represent an ancient adaptation to oxidative stress, rather than a system evolved primarily for generating genetic diversity (Hörandl and Hadacek 2013).

Speaking of the harmful effects of ROS, one of the most significant effects on meiosis is the premature loss of sister chromatid cohesion in prophase I. The meiotic cohesin complex, composed of structural maintenance of chromosomes proteins SMC1 and SMC3, and meiosis-specific α-kleisin REC8/SYN1 proteins (Hsieh et al. 2020). The complex, with the cooperation of ECO1 (ESTABLISHMENT OF COHESION1) acetilase, maintains physical connections between sister chromatids throughout meiotic prophase I (Bolaños-Villegas 2021). Research conducted on *Drosophila* oocytes demonstrated that knockdown of the ROS-scavenging superoxide dismutase (SOD) enzymes leads to significant increases in chromosome segregation errors (Perkins et al. 2016). The vulnerability of cohesin to oxidative damage is particularly high in arm regions of chromosomes compared to centromeric regions. FISH analysis of Perkins and colleagues (2016) revealed that SOD knockdown increases the percentage of oocyte chromosome arm cohesion defects. This then leads to destabilization of chiasmata and increased frequency of recombinant homolog mis-segregation during meiosis I. Mutants of cohesin subunits and ECO1 showed increased oxidative stress sensitivity in budding yeast (Mfarej and Skibbens 2022) providing further support on the contribution of these proteins to oxidative stress derived defects of meiosis. In plants, manipulation of cohesin components offers molecular breeding opportunities. First, modulation of REC8/SYN1 expression can alter CO frequency and distribution to lower linkage drag. Second, the optimization of the balance between cohesin stabilization (ECO1/CTF7, SWI1 genes) and release (WAPL gene) can improve plant tolerance to chronic DNA damage generated by oxidative stress (Bolaños-Villegas 2021). Third, designing weak wapl alleles or PCNA-ECO1 fusion modules may facilitate repair under replication stress in PMCS, although fertility may decrease (De et al. 2016; Liu et al. 2020). CRISPR-based approaches targeting cohesin components may optimize unreduced-gamete frequency while maintaining normal fertility in non-target generations, thereby accelerating DH production cycles in maize, wheat, and rice breeding programs (Kuo et al. 2021; Li et al. 2025a, b). Simulation studies exploring controlled elevation of meiotic recombination frequency - via suppression of anti-CO factors such as RECQ4, or through overexpression of pro-CO factors such as HEI10 - have been conducted in wheat (Taagen et al. 2022). The authors discouraged the controlled recombination approach as a practical breeding strategy -at least in the case of hexaploid wheat- noting that prediction accuracy consistently declined under increased recombination when using actual marker data (rather than perfect QTL knowledge), and that generating desired multi-site crossovers simultaneously across all 21 wheat chromosomes carries a probability of approximately 1/2²¹ - effectively impossible in practice (Taagen et al. 2022).

The ATM kinase is a central coordinator of the cellular response to oxidative DNA damage, from prophase DSB repair to anaphase progression. Inactive ATM homodimers are activated directly by ROS or indirectly via the MRN (MRE11-RAD50-NBS1) complex (Cosentino et al. 2011). Activated, monomeric ATM then phosphorylates H2AX at DSB sites and also activates checkpoint pathways (Cosentino et al. 2011; Zhang et al. 2018). The ATM protein plays an important role during plant meiosis, as it coordinates the formation and repair of programmed DSBs (Garcia et al. 2003; Zhao et al. 2023). These meiotic DSBs are repaired by homologous recombination, after the recruitment of the MRN complex. These complex processes break ends for strand invasion, preferentially using the homologous chromosome as template over the sister chromatid (Keeney et al. 1997; Zhao et al. 2023). ATM mutants are partially sterile, due to chromosome fragmentation during meiotic anaphase, highlighting the role of the gene in the defense of meiosis against oxidative harm (Garcia et al. 2003).

Meiotic recombination in plants relies on SPO11-dependent DSBs for initiation, but oxidative stress also generates SPO11-independent DSBs. However, evidence for direct utilization of ROS-induced DSBs as templates for meiotic recombination is still limited and indirect. In *Coprinus cinereus* (a fungal model relevant to plant studies due to shared meiotic system), a nbs1 mutation leads to replication-derived DSBs that support Spo11-independent crossovers without apparent Spo11 dependent DSB induction (Crown et al. 2013). These replication-dependent DSBs may include lesions influenced by endogenous oxidative stress, although ROS involvement has not been directly demonstrated in this model.

ROS, as discussed above, cause oxidative DNA damage including DSBs in plant cells, including meiocytes. Oxidative mutagens like rose bengal increase somatic HR frequency in *Arabidopsis* (Filkovski et al. 2004). This suggests that ROS-generated lesions are repaired via HR pathways that share core components with meiotic recombination (Filkowski et al. 2004; Manova and Gruszka 2015). However, these occur in somatic or mutagen-treated contexts and not verified during native meiosis. Current evidence shows that plants prioritize SPO11-dependent DSBs for recombination which ensure chiasmata and proper segregation (Wang and Copenhaver 2018).

During prophase I, pesticide-driven ROS production can also enhance cytomixis. Siddiqui and Alrumman (2022) reported that various pesticide treatments in Pisum sativum pollen mother cells triggered dose-dependent cytomixis and syncytes through redox imbalance and cytoskeletal instability. This shows how excess ROS disrupts meiotic stability, linking oxidative damage to inter-PMC chromatin migration beyond natural abiotic stressors.

In later meiotic stages, ROS-induced oxidized bases and DSBs compromise chromosome stability. This form of DNA damage often induces major rearrangements in eukaryotic model organisms (Vanderauwera et al. 2011; Szurman-Zubrzycka et al. 2023). Similarly, chronic oxidative stress frequently induces chromosome missegregation and the formation of micronuclei in plant cells (Kwasniewska and Bara 2022). Rupture of micronuclear envelope and continuous exposure to ROS then leads to faulty repair and chromothripsis-like shattering (Xu et al. 2014; Di Bona et al. 2024). Excess ROS generation in meiotic cells weakens chiasmata and cohesion and paves the way for nondisjunction and aneuploid gamete formation, as seen in superoxide dismutase-deficient Drosophila oocytes (Perkins et al. 2016, 2019). Dense DSB clusters, probably containing ROS triggered lesions, seem to be in the background of chromoanagenesis. This catastrophic event happens when several chromosomal rearrangements occur simultaneously in one or a few chromosomes, leading to extensive localized genome restructuring (Guo et al. 2023). Chromoanagenesis typically originates from non-homologous end-joining (NHEJ) or microhomology-mediated break-induced replication (MMBIR) on single chromosomes (Guo et al. 2023; Samach et al. 2023). According to recent literature, ROS acts as both genotoxin and modulator of DNA damage response (DDR) networks, driving cells toward genome restructuring (Nisa et al. 2019; Cimini et al. 2019). Oxidative stress induces telomere dysfunction and shortening in all cells, both in plants and animals (Kordowitzki 2021; Castillo-González et al. 2022). The high guanine content of telomeric regions makes telomeres particularly susceptible to oxidative damage, as here there is high chance for the formation of 8-oxoguanine lesions and single-strand breaks (Kordowitzki 2021). The resulting telomere dysfunction can trigger apoptosis and enhance chromosomal instability, as well as aneuploidy formation during meiosis (Hemann et al. 2001). These effects are especially important in non-dividing cells with long lifespan (such as aging human oocytes), where chronic ROS leads to progressive telomere shortening (Kordowitzki 2021). In plants, however, telomere malfunction does not certainly trigger rapid cell death. For instance, *Arabidopsis* telomerase knockout mutants remain viable for several generations despite progressive telomere shortening and severe developmental abnormalities (Riha et al. 2001; Shakirov et al. 2022). Moreover, while null mutations in the CST capping subunit CTC1 cause considerable telomere shortening, the carrying plants are viable, despite the occurring growth arrest and sterility (Surovtseva et al. 2009; Shakirov et al. 2022). *Arabidopsis* mutants with exceptionally short or long telomeres show altered reproductive fitness and sensitivity to genotoxic and other abiotic stresses. This points to the fact that telomere status influence stress responses rather than only setting a threshold for viability (Riha et al. 2002; Bundock et al. 2002; Campitelli et al. 2022; Shakirov et al. 2022). Taken together, although oxidative stress-induced telomeric damage is indeed a common feature of plant and animal cells, direct extrapolation from animal models to plants is problematic, as plants can tolerate a considerable degree of telomere dysfunction (Campitelli et al. 2022; Shakirov et al. 2022).

#### Molecular responses and defense mechanisms

Plants have evolved sophisticated antioxidant systems to combat oxidative stress in all cell types and during all developmental phases, including meiosis. The superoxide dismutase (SOD) protein family have isoforms with different compartment localization (reviewed by Alscher et al. 2002; Mittler 2002). However, in severe stress conditions, SOD activity can be decreased by direct ROS damage or by regulatory mechanisms (Guan et al. 2013). Catalase enzymes serve as critical H₂O₂-scavenging components, particularly in peroxisomes, where photorespiratory H₂O₂ is produced (reviewed by Mhamdi et al. 2010). In rice anthers, rapid inhibition of OsCATB catalase transcripts during heat stress contributes to ROS accumulation and ultimately to pollen sterility (Zhao et al. 2018). Ascorbate peroxidase enzymes are present in multiple cellular compartments and work coordinately with the ascorbate-glutathione cycle to maintain redox homeostasis (reviewed by Shigeoka et al. 2002; Maruta et al. 2016). Glutathione peroxidases (GPXs) effectively reduce both H₂O₂ and organic hydroperoxides (Chen et al. 2004; Navrot et al. 2006; Islam et al. 2015). Plant GPXs show differential expression during exposure to various stress conditions and maintain cellular redox balance (Milla et al. 2003; Islam et al. 2015).

#### The dual nature of ROS: signaling versus damage

A critical but inadequately addressed gap in our knowledge in meiotic oxidative stress research is distinguishing physiological ROS signaling from pathological oxidative damage. Accumulating evidence demonstrates that ROS function as essential signaling molecules during normal reproductive development, with precise spatial and temporal control of ROS production being crucial for male and female gametogenesis (Ali and Muday 2024). The biphasic dose-response relationship of ROS-beneficial at low concentrations but detrimental at high levels-has been documented in mammalian oocytes (Calabrese et al. 2024) still uncharacterized in plant meiosis. This holds a fundamental problem: when environmental stress elevates ROS levels, are we observing the disruption of essential signaling pathways or the imposition of oxidative damage on vulnerable cellular structures? The answer likely varies with ROS concentration, cellular compartment, and developmental timing. Transition from physiological to pathological oxidative states should be studied in the future to dissect the biphasic nature of ROS. Researchers already have tools, such as organelle-specific ROS sensors and real-time imaging to use on meiocytes for this purpose (Akter et al. 2021). Without sufficient knowledge, genetic engineering to alleviate oxidative stress may disrupt critical ROS signaling, and strategies used to preserve ROS signaling might fail to prevent oxidative damage.

## Conclusions and future perspectives

**Fig. 2:**
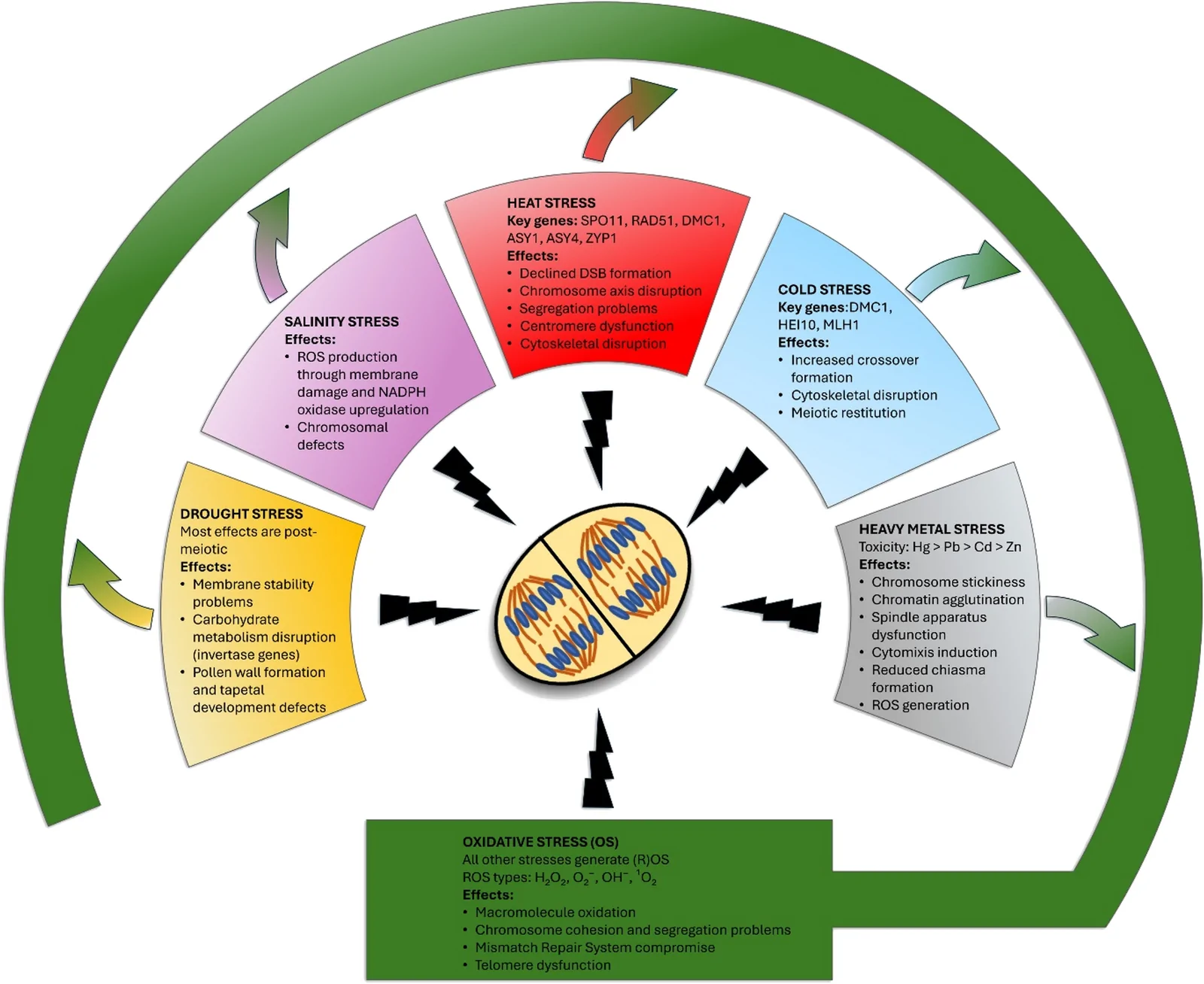
Effects of various environmental stresses on the meiotic process. Heat, cold, drought, salt, and heavy metal stress create oxidative stress through ROS production, and also significantly disrupt different aspects of meiosis individually

As demonstrated above, environmental stresses present critical and diverse threats to plant meiosis (for summary, see Fig. 2). The stress sensitivity of meiotic division, achilles’ heel of plant sexuality limits fertility in many plant species. The persistence of these weaknesses despite strong selection for reproductive success presents a genuine evolutionary paradox. Several non-mutually exclusive explanations can be proposed.

The meiotic machinery is highly conserved and constrained by pleiotropic effects. Evolutionary flexibility of proteins having multiple functions in meiotic chromosome organization, DSB formation, and repair is severely limited (Bomblies et al. 2016). However, some of the meiotic weaknesses carry also adaptive features, as increased recombination frequency and altered crossover distribution could improve adaptation effectiveness (Phillips et al. 2015; Lloyd et al. 2018). Plants have been experiencing repeated whole-genome duplications throughout their evolutionary history, where polyploidy serves as a major driver of speciation and adaptation (Otto and Whitton 2000; Vlček et al. 2025). Weak checkpoints that permit completion of meiosis despite chromosome pairing problems may facilitate the establishment of nascent polyploids, which would be eliminated in systems with strict surveillance mechanisms (Bomblies et al. 2016). The plant tolerance for aneuploid gametes and zygotes differs markedly from animal systems. Although aneuploidy generally reduces fitness, plants can tolerate chromosomal imbalances, with selection acting in dosage-dependent and also in chromosome-specific ways (Huettel et al. 2008; Henry et al. 2009). This inherent flexibility has important evolutionary implications. It allows plants to explore chromosomal variation space more extensively than would be possible under stringent selection against aneuploidy. Plant meiosis operates in a state of balanced vulnerability. Although accurate chromosome segregation is important for reproductive success, plants accepting certain errors may gain evolutionary advantage. This duality originates from the evolutionary history of plants: as sessile organisms, they respond to environmental perturbations via plasticity rather than avoidance. Consequently, meiotic weaknesses may be better understood as inevitable constraints of mechanistic complexity of the process and trade-offs with polyploid evolution, rather than failures of optimization.

A review of the meiotic stress literature reveals a clear predominance of studies on the male side. This bias originates from the much higher experimental accessibility of microsporogenesis and pollen development (Lambing and Heckmann 2018; Kuo et al. 2021). In contrast, female meiosis and its long-lived products seem more susceptible to cumulative genome damage, particularly at chronic oxidative or genotoxic stress. Overall, research data suggests that male meiosis fails more often at acute abiotic stress, while its female counterpart may be relatively more buffered against absolute failure. However, female meiosis is still highly sensitive to relatively minor defects regarding recombination, cohesion, and genome integrity, resulting in aneuploidy and altered mutation spectra in the offspring (Phillips et al. 2015; Saini et al. 2017; Perkins et al. 2019; Zhao et al. 2023). It would be important to study stress impacts on both male meiosis and female meiosis, rather than concluding from male meiosis data alone (Lambing and Heckmann 2018; Kuo et al. 2021; Chen et al. 2025).

While cell biology, genomics and transcriptomics continuously broadens our knowledge on the sensitivity of meiotic apparatus, there still remain areas to be explored. While epigenetic regulation of meiosis is an emerging field (Chowdary et al. 2023), additional data regarding transgenerational aspects of meiotic stress response would be important. Revealing the alterations in non-coding RNA expression, the post-translational modifications and variants of histone proteins, as well as DNA methylation changes, induced by environmental stress during meiosis may open new opportunities for breeders to create crops with improved resilience (Gallusci et al. 2023). As meiosis represents a highly dynamic system, it is also essential to thoroughly study meiotic phase transitions and that regulate the progression of the process. Nevertheless, precise application of stress and assessment of reactions and recovery mechanisms may be challenging tasks for investigators. In addition, complex polygenic base of meiotic stress tolerance demands comprehensive gene expression studies that readily capture epistatic interactions and genotype-by-environment effects across stress factors.

It is fundamentally important to translate the results of meiotic stress studies into solutions for successfully addressing challenges facing crop production. Breeding efforts need to focus on the improvement of meiotic stress resilience in the future to meet this challenge. Temperature-sensitive meiotic genes (e.g. DMC1, CENH3, HSP101, TAM/CYCA1;2) are potential key targets for the development of climate-resilient crop varieties. Antioxidant defense systems also require enhancement in crop plants to combat oxidative stress-induced meiotic failures. As climate change and increasing human impacts on our planet continuously tests crop resilience, knowledge and manipulation of meiotic stress responses become more important than ever for global food security. The combination of modern molecular tools, precise phenotyping systems, and systems level strategies offers new opportunities to produce crops that possess stronger reproductive tolerance.

## Data Availability

Not applicable.
